# Obstetrics, Etc.

**Published:** 1857-01

**Authors:** 


### OBSTETRICS, &c.

1. Carbonic Acid as a Local Anæsthetic in Uterine Diseases, &c.—Prof. Simpson made some remarks on this, at a recent meeting of the Obstetrical Society of Edinburgh. He said that he had used carbonic acid successfully, as a local anæsthetic, in neuralgia of the vagina and uterus, and in various morbid states and displacements of the pelvic organs, accompanied with pain and spasms. He had found it also sometimes of use in irritable states of the neighboring organs. Two years ago, he had under his care, from Canada, the wife of a medical gentleman, who was suffering much from that most distressing disease,—dysuria and irritability of the bladder. Many modes of treatment had been tried in vain. The injection of carbonic acid gas into the vaginal canal several times a day, at once procured relief, and ultimately effected a perfect cure. She has remained well since her return to America, and lately became a mother. Occasionally, relief follows immediately. In two or three instances, he stated he had seen the use of the gas continued daily for months, and that he had notes of one case where the patient was almost invalided, and almost entirely kept to the supine posture, for years, from feelings of pain and bearing down in the uterus and neighboring parts, particularly on attempting to sit or walk. Many modes of treatment were tried by himself and others, with little or no benefit. She has, however, at last regained in a great measure the power of progression, and freedom from suffering in the erect posture—a result which she herself ascribes to the local application of carbonic acid gas.

In practice, he generally used a common wine-bottle for the formation of the carbonic acid gas, and formed the gas by mixing in the bottle six drachms of crystallized tartaric acid with a solution of eight drachms of bicarbonate of soda, in six or seven ounces of water. A long, flexible caoutchouc tube conducts the gas from the bottle into the vagina. The cork fixing this tube into the mouth of the bottle should be adapted so as to prevent any escape of the gas by its sides. With this view, the cork should be perforated by a metallic tube, and covered externally with a layer of caoutchouc. In a case in which the two preceding children were both lost, he had successfully brought on premature labor at the eighth month, by the repeated application of carbonic acid gas to the vaginal canal with this apparatus—the carbonic acid not acting directly as a specific oxytoxic or excitor of uterine contraction, but indirectly only, by distending greatly and mechanically (as examination with the finger proved it to do) the vaginal canal, and ultimately separating, like the injection of water, the membranes from the cervix uteri.

The application of carbonic acid as a local anæsthetic to the uterine mucous surfaces, and to other parts of the body, is not a discovery of late times. He had found that in this, as in many other examples, that what appeared at first novel, was, when fully investigated, a practice known previously in its essence, and perhaps in its more minute details also. Besides, here as elsewhere, when once a principle is detected, such as the anæsthetic power of carbonic acid gas when applied topically, we can explain by it the good effects of modes of practice which previously, perhaps, we were inclined to ridicule and reject. The fact that carbonic acid, when locally applied to a mucous surface, acts as a sedative or anæsthetic, explains a practice common among the ancients, viz., Hippocrates, Paulus Ægineta, Rueff, Paré, etc., all of whom used to burn herbs, aromatic and medicinal, and convey the fumes, by means of tubes and appropriate apparatus, to the interior of the vagina; and, such vapor being loaded with carbonic acid, it is more than probable that if such treatment was effectual, it was through the anæsthetic properties of the gas here alluded to. Again, there is a modern practice much in vogue on the Continent, of injecting the vagina, etc, with the German waters of Nuheim, Marienbad, etc.; the utility of the practice, which Dr. S. has been assured by his friend, Dr. Funck, of Frankfort, is most marked in some diseased states, will find its true explanation in the local anaesthetic effect of carbonic acid, as these waters contain a large quantity of the gas. A knowledge of the topical effects of carbonic acid serves, perhaps, also to afford an explanation of other points in common therapeutics; as, for example, its action in subduing gastric and intestinal irritation. Hence the use of effervescing draughts, aerated waters, etc., in gastric irritability and nausea; perhaps the antacid action of the alkali may have some effect, but most likely it is the anæsthetic properties of the carbonic acid gas. The sedative and curative effects of injections into the rectum of carbonic acid gas in dysentery have a similar explanation, and serve to corroborate this view of its action. As an example of its use as a local anæsthetic to a cutaneous surface, Dr. S. alluded to the *cataplasma cerevisiæ,* or yeast poultice, which exhales from its surface a quantity of the gas. It was commonly applied to irritable and sloughing sores, and its soothing, healing, and antiseptic properties were doubtless owing to the carbonic acid gas. As an anæsthetic application to cancerous ulcers, the effects of carbonic acid gas are excellent. Dr. Ewart, of Bath, says, "he has kept a person in ease and comfort, who, for so great a length of time before, had known only agony and torture." "What," he elsewhere observes, "strikes us in the two preceding cases with the greatest astonishment, is the *almost instantaneous relief of pain* which *never failed to follow the application of the gas.*" In reference to the effects of carbonic acid upon raw surfaces and wounds, Dr. Ingenhouz mentioned to Beddoes the following experiment: "Blister your finger, so as to lay bare the naked and sensible skin. The contact of air will produce pain; put your finger into vital air (oxygen), and this will produce more pain; introduce it into fixed or azotic air (carbonic acid or nitrogen), and the pain will diminish or cease." In relation to this statement, Dr. Beddoes informs us that he made the following experiments on three different persons: *First.* The raised epidermis of a blistered finger, after all action from the cantharides had ceased, was cut away in carbonic acid gas. No pain was felt. *Secondly.* A second blister being opened in common air, smarting pain came on. In a bladder of fixed air, this pain soon went off. *Thirdly.* After opening a third blister, the finger was instantly plunged into oxygen. It felt as when salt is sprinkled on a cut. In carbonic acid gas, the pain, in two minutes, quite subsided; but returned when the denuded skin was again exposed to the atmosphere. If there be no source of fallacy in these experiments, they certainly point to one kind of improvement in the treatment of some painful burns, wounds, etc.; for they appear to suggest the possibility of the suffering which is attendant on such injuries being controlled and cancelled by keeping the pained parts in contact with carbonic acid, or with some other gas or fluid capable of acting as a local anæsthetic. If the reports of Ewart, Beddoes, and Fourcroy are correct, we ought, also, indeed, to find carbonic acid an excellent application even as far as the mere healing and cicatrization of the broken surfaces are concerned.—*Edinburgh Med. Journ.*, July, 1856.

2. EFFECT OF CARBONIC ACID ON THE GRAVID UTERUS.—The *Union Medicale*, for August 12, contains an account of a case in which carbonic acid gas, injected into the vagina, was successfully employed to effect premature delivery. The gas was generated in a glass vessel, by means of the action of acetic acid on the bicarbonate of soda, and conducted into the vagina by means of an elastic tube. A glass speculum was introduced into the vagina, and in order to prevent the carbonic acid from escaping too freely, the tube was passed through a cork which closed the external opening of the speculum. The injection of the acid was followed by a disagreeable pricking sensation in the vagina. After the third application, some pain was felt in the umbilical region, and the cervix uteri became softened. Six applications were made, of from twenty to thirty minutes each, one at night and one in the morning; after the last one, uterine contractions came on, and delivery followed. The result of this case is very satisfactory, so far as it goes, and is worthy of trial in cases where it becomes necessary to procure premature delivery, especially as it occasions no harm either to mother or child, and, at the worst, only causes the delay of a few days.—*Boston Med. and Surg. Journ.* *Dec.* 4, 1856.

3. CARBONIC ACID AS A MEANS OF INDUCING PREMATURE LABOR.—Scanzoni, moved by the observation of Brown-Sequard, that carbonic acid irritates the smooth muscular fibre to contraction, and convinced of the insufficiency of his method of exciting labor by suction of the breast, has sought in carbonic acid a new means of exciting labor-pains. In a very small primipara, aged twenty-six, premature labor was indicated by contraction of the pelvis. She was in the 32d-34th week of gestation. The portio vaginalis was five to six inches long, tolerably resistant; outer os uteri fast closed; the head floated; the fœtal pulse faintly heard. On the 2d February, the first application of twenty minutes without subjective or objective alteration.

3d February, eight A. M., for twenty-five minutes, and in the evening, thirty minutes. During the injection, pricking in the vagina; during the day often stinging in the region of the umbilicus; in the evening the portio vaginalis was loosened. The stingings were renewed in the night.

4th Feb. Morning and evening, each time half an hour. Pricking in the vagina. In the course of the day, the os uteri admitted the finger through, and the presenting head could be reached. In the night labor-like pains, and towards morning lively contractions of the uterus, which latter ceased.

5th Feb. Pricking during the thirty minutes of the injection. The os was opened, yielding, dilatable. Increased vaginal secretion. About noon, painful persisting contractions; about half-past six P. M., rupture of membranes; seven P. M., birth of a living child over three pounds weight. Slight metrorrhagia, which ceased after the removal of the placenta. Recovery good.

*Apparatus.*—A glass vessel, holding a quart, is fitted with an air-tight cork-stopper, in which are two openings. Through one opening runs a glass tube provided with a funnel; through the other runs a horn tube fitted with an elastic tube three feet long, which ends in a bent uterine tube. Two tablespoonfuls of bicarbonate of soda, dissolved in twelve ounces of water, and a little vinegar, serve to supply the carbonic acid. A conical glass speculum and the uterine tube are introduced into the vagina, the tube being surrounded by a cork filling up the speculum, so as to retain the carbonic acid in the vagina.—*Brit. and For. Med.-Chir. Rev.*, Oct., 1856.

4. STRICTURE OF THE UTERUS.—" Dr. Lehmann, of Amsterdam, gives a minute analysis of the forms of irregular uterine contraction, under the name of stricture of the uterus. Stricture of the uterus, he says, a partial tonic spasm, happens almost exclusively in the direction of the transverse fibres, especially in those places where the circular fibres predominate, at the lower part of the body of the womb, at the os internum and os externum uteri, and in the neighborhood of the Fallopian tubes.

" Tonic spasm of the lower part of the body causes a ring or band-like contraction more or less broad, which, when well developed, can be felt through the abdominal walls. The uterus seems to be irregularly shaped lengthwise, reaches to the pit of the stomach, and is often divided into two unequal halves, so that the lower half is separated like a strongly-distended urinary bladder. The introduction of the hand makes the diagnosis clearer; mostly, a small, smooth, defined line, stronger in front, can be felt. The spasm-affected part is very painful to the touch, and the pain, as well as the contraction, remains beyond the labor-pain. The conclusion is erroneous, that these strictures happen only in the fifth stage; they are observed nearly as often in the third, and frequently they arise first when the head and shoulders are behind, or when, in a breech presentation, the breech is partly born. This condition is known by the following marks: in spite of strong pains the presenting part recedes, and although no obvious obstruction exists in the pelvis. When the head presents, it is found that the pains do not drive it down on the os uteri, showing that the obstruction does not lie here, but higher up. If, in such a case, through wrong diagnosis, forceps be used, the blades pass readily through the os uteri, but in pressing deeper strike upon an obstacle which cannot be overcome without great pain. If the application of the forceps be accomplished, it is found on each extractive effort, that the uterus is dragged down too. If turning is tried, the stricture is much more easily recognized. The hand easily penetrates the cervix uteri, but the fingers are with difficulty squeezed between fœtus and uterus, and if the whole hand be passed through, it is quickly paralyzed by the compression. The stricture is more easily known in the fifth stage, when the fingers may be passed to the contracted part, and the hour-glass form recognized.

The *spasmodic stricture of the inner mouth of the womb* is most frequent in the fifth stage, but rare during the extrusion of the child. It is recognized by the same signs, only nothing can be felt through the abdominal walls; on the other hand, it is easily detected by vaginal examination. The os uteri externum may be flaccid and widely open, without trace of contraction.

The *spasmodic stricture of the os uteri externum* is the least dangerous form. Early in labor it is clear to the observer that the pains are irregular and painful, the patient is restless and uncomfortable. The strong boring pains are felt, chiefly deep in the hypogastrium, and spread towards the sacrum and thighs in the course of the ischiatic nerve. The vagina is mostly hot, dry, and sensitive; the os uteri remains deep in the pelvis, and has very thin sharp edges, which, even in the interval of contraction, are stretched like a cord. The presenting part lies firmly on the lower segment of the womb, and even if a little engaged in the os uteri, a large swelling is caused by the pressure of the stretched edge. The sphincters in the neighborhood often share the spasm, and dysuria and tenesmus follow; or there arises, through the extension to the other nerve-spheres, hiccough, vomiting, cough, anxiety, and oppression, in the highest degree; the central nervous system is even affected; syncope, headache, delirium, sopor, convulsions, whilst, although the pains may continue, what is called metastasis takes place.

The *spasm of the mouths of the Fallopian tubes* only occurs in the placental stage. The womb assumes a remarkably oblique shape, as if the affected part had been lengthened out like a horn, as can be felt as well as seen.

Uterine strictures, when they occur, must be regarded as consequences of irregular contraction; but they may exist, according to Lehmann, as a real alteration of tissue at the affected part; this may have arisen before the beginning of the stricture, or may follow upon a long-continued contraction, through which stasis in the uterine vessels, hyperæmia and inflammation of the whole organ may be easily developed. Not seldom it is observed that an ordinary clonic spasm passes gradually into a stricture, especially in the lowest part of the womb. Still more frequently it happens that, in a clonic spasm, at the time when the child is extruded, a uterine stricture follows in the fifth stage. But the predisposition to stricture often rests in the uterus, as in hyperæsthesia, through a rheumatic or inflammatory action, when an alteration of tissue may have existed during pregnancy. Besides this, strictures may arise, or become worse, through bad presentations of the child, after premature escape of the liquor amnii, through irritation of the os uteri from the frequent exploration, or instrumental interference.

Stricture of the uteri always perverts the course of labor, but naturally according to the degree, duration, seat, and stage of labor. The effect of the compression is also severely felt by the child. Bruises are sometimes seen, and asphyxia; the liver has been known to have burst. The uterine walls may be rent.

Lehmann advises to bring about retraction of stricture by venesection, opium, warm baths, belladonna injections. He has found ether and chloroform without influence.—*Nederl. Zigdschr.*—*Ibid.*

5. CRIMINAL ABORTION—PERITONITIS.—At a recent meeting of the New York Pathological Society, Dr. T. C. Finnell presented the uterus removed from a woman upon whom abortion had been produced, and who subsequently died of *peritonitis*, with the following account:

Matilda McCarty, aged nineteen, being about four months advanced in gestation, was desirous of having abortion produced. She was advised to use a vapor bath of pennyroyal and tansy, and remain in the bath at least twenty minutes, then walk to Forty-second Street and back again to her residence, in Spring Street, as quickly as possible; also to drink freely of gin and tansy before leaving the house. This was on the 7th of March. On returning from her walk, she complained of great distension of the abdomen by flatus, inability to walk, and a feeling of great prostration. At the end of a week, labor pains came on, and she was soon delivered of a dead fœtus. On the third day after delivery, she seemed well enough to be up. At the end of the week she was seized with a chill, followed by fever, which terminated in death, on the 28th of March, three weeks after delivery. The *autopsy* showed extensive peritonitis, with adhesion of all the abdominal organs to each other. About a pint of pus was in the pelvis, surrounding the uterus.—*N. J. Med. and Surg. Rep.*, Oct. 1856.

6. ON THE DEVELOPMENT AND DECLINE OF THE FUNCTION OF MENSTRUATION WHEN NOT IN CORRESPONDENCE WITH THE GENERAL SYSTEM. By *James Allen, M.R.C.S.*—The phases of disease are so varied, that sometimes, by changing our stand-point, we extend our survey, and bring out different combinations, which may help to more distinct comprehension of the laws which regulate life and health. The following observations, on a class of diseases which are very common, I trust may prove interesting, as contemplated from a new point of view.

It is an undoubted fact, that the growth or development of parts is by no means uniform, regular, and consentaneous; and that, while we have clear and distinct laws of animal growth, in which we see at various periods the regular development of parts, and the organs by degrees becoming adapted to their functions, yet these are by no means uniform in their manifestations. It would form an interesting inquiry to ascertain how far these varieties, in their different combinations, influence the health of the subject; also, how, by their correspondence or incongruity, they give rise to varying phenomena.

In childhood we have fewer discrepancies than in youth; still, now and then, we meet with marked cases both of deficiency and precocity; in some, decided excess in the circulation, tending to affections of excitement; in others, defective powers of assimilation; in some, defective formation of bone, yet with no lack of muscular power; in others, power with no apparent will, etc. In youth, these examples are more palpable; here we may see growth in excess, generally without strength to resist external influences, and the subject succumbs to disease; excessive prolongation of limb, with narrow chest, or feeble digestive power, whereby the balance of health is soon destroyed. But the most marked are, where we have interesting cerebral development, at the expense of disease or deformity. We may have many examples of want of correspondence of organs in their development, until the subject arrives at that period when it may be said to have attained maturity.

The organs involved in the functions of menstruation are liable to this irregularity; but passing those extremes that partake more of monstrosities, I would notice such as occur about the ordinary periods when the uterine system does not correspond with the general system. Without attempting to particularize all the causes in which they originate I may mention climate, habit, occupation, social and domestic circumstances, and individual idiosyncrasies, or some morbid action retarding general or particular development.

We will consider those in which the general system takes the precedency, and, in our climate, I believe this to be the most frequent. Here we have the aspect of the woman with much of the artlessness of the girl, wanting the shyness so peculiar to puberty; good health is enjoyed until that period arrives when the system is so advanced as to require the call made upon it by the uterus in menstruation, but, development being incomplete, the system soon begins to feel the burden, and then we have a variety of affections. Diagnosis is here difficult, yet all-important; but where we can trace regular development up to a certain point, without the signs of puberty, then we must watch the character of the first aberration of health, and we shall find that slight but decided determination to some part, with excitement, will be the result; afterwards more decided changes appear, the plethoric condition of the vascular system in many is very prominent, and we have hemorrhage, active or passive congestions, inflammations, etc. Where there is a strong constitutional diathesis there is danger of active disease being set up; and here, for practical purposes, it is of great importance to distinguish cause from effect, and to apprehend clearly how morbid influences act and react, lest we should prescribe for symptoms merely, and overlook the first link in the chain. Many strumous affections have here their origin, in organs or structures where congestion first sets up morbid action, and then, in some instances, this reacts on the system to suspend or arrest further development. Therefore, in treatment we must be careful not to stop the regular development in our efforts to relieve the oppressed organs; when both have due attention we shall best succeed. There is one practical consideration needful in all cases, and that is, when the morbid action does not relieve the system to the right extent of the burden with which it is surcharged; then our object should be to assist nature, and this may be accomplished best by small bleedings, from a few drachms to two or three ounces, and this repeatedly; and, when we have succeeded thus in arresting tendency to disease, we must relieve the system by small abstractions periodically, until nature relieves herself.

Under such combinations I would suggest a caution as to stimulants, or iron as an emmenagogue, lest we should further depress some oppressed organ by attempting to excite uterine discharge. In these examples of lack of correspondence, according to my experience, nothing equals small repeated bleedings, with very mild aperients.

When we meet with cases the reverse of these we have just noticed, where the uterine system takes the precedence, we have all the changes common to womanhood in manner and bearing, with mammæ developed and other signs of puberty; but this generally with a stinted growth, and a puny and sickly aspect. The subject is characterized by great nervous sensibility, and is easily depressed both in mind and body. Here neuralgia in various forms is common, inflammations rare, and, if there is fever, it assumes a low type; not many strumous affections as the result, though sometimes spinal complaints. The regular menstrual discharge, so often hailed by mothers, is here an evil; and, while we should use no direct means to stop this, yet, if tonics and astringents do so, it is generally to the advantage of the patient; regular sluicing, with cold salt and water, is very beneficial, and whatever will invigorate and support the system should be adopted. I have a high opinion of malt liquor in such cases, not merely at meals, but given at stated intervals in measured quantities, as ordinary tonics. Cod-liver oil is here of great value; iron is questionable, but quinine and acids seldom wrong. All that can be done by nutritious diet must be attempted, with pure air and change of place, when convenient. The great evil such cases exhibit is, that generally a stop is put to more perfect development, and then we have illustrations of ill-formed, disproportioned figures, for which I know no remedy.

The decline of life, like growth, has its irregularities as to degree in different organs; and, could we more easily distinguish where they first fail, quite independent of the influence of accident or disease during life, we should frequently be the better able to advise wisely for our patients. In some instances we see the digestive organs first fail; in others, the lungs, the heart, or the head—all corresponding to the irregularities of development, which we would do well more carefully to investigate.

I would now call attention to a few of the circumstances which affect women on the decline of menstruation, when the uterine and the general systems lack correspondence. Without attempting to generalize, I would merely give what has more immediately fallen under my own observation. And, first, I would remark, that I have seen more cases in which the uterine organs have first declined in power, than the system generally. In these examples we have a great variety of disorders; yet, when clearly distinguished as to origin, they are very manageable. The characteristic fact under these circumstances is, that the system becomes oppressed from a surcharge on the circulation, which the uterine organs can no longer carry off—then plethora, local or general inflammations, or congestions, are the common result. Much has been written or said about the displacement and inflammatory theory, but I am persuaded that many of these disputed cases have their origin in hypertrophy, consequent upon the very condition just supposed; and congestion, not inflammation, produces these changes, which, when duly traced, will be found thus to arise; and the origin indicates the treatment. And here I hesitate not to affirm, that nothing will relieve the patient like regular periodic bleeding, and that, not necessarily from the os uteri, but, when leeches are employed, just within the labia pudendi; if by the lancet, two or three ounces every three weeks will do more than all other means combined; in fact, artificially unburden the system, and *vis medicatrix naturæ* will rectify all the evils if they have not been persistent too long. Functional derangement consequent upon this condition is most successfully treated by repeated bleedings to small amount, especially when the head and stomach suffer.

Another class of ills have here their origin, but are very different in their character; and mistakes, I believe, are common, viz., where you have repeated, irregular, large discharges of blood, as menstruation declines, from the fact of the uterine organs so failing in power as not to accomplish their healthy and due discharge; but from loss of tone the vessels are not able to bear the ordinary congestion and strain upon them, but yield irregularly, and pour out large and abnormal quantities, rendering them less able to resist in the future; and in many instances the patient is safe only when the system has been thus brought down to the level of the feeble uterine organs. In such cases a clear apprehension of the condition points to the treatment. These failing organs, when the subject of repeated determinations, no doubt often have degeneration of tissue, and this lays the foundation for organic disease. These large discharges differ from ordinary catamenia, not only in character, but in quantity, and especially in time. These combinations are, I believe, the source of most uterine diseases met with at this period of life; for, according to my experience, the continuance of menstruation beyond the due period is fraught with many ills to the system generally, but few to the organs themselves.

In examples where we meet with decline of the general powers of the system, the uterine being entire, we have the subject put on the appearance of premature old age, weak, languid, and rapidly altered aspect; very nervous, but few cases of inflammation, or acute forms of disease. To sustain the system must be our object; cold bathing seldom answers, but change from place to place is very decidedly useful.

I have thus attempted briefly to point out some of the disorders consequent on the want of correspondence between the general and uterine systems, and believe in this way we get a solution of many of the phenomena attendant thereon, as well as having the best treatment indicated; I also feel assured this view assists us in the better understanding of many of the abnormal states of menstruation, at all periods, as, amenorrhea, dysmenorrhea, menorrhagia, sterility, etc.

All must admit "that, to arrange under new combinations what is already known to us, is often in itself a source of fresh knowledge, or a valuable means of correcting previous error."

In conclusion, I would express a hope that this mere outline may be suggestive to some to bring out more prominently all the features and combinations presented by this interesting class of affections.—*Med. Times and Gazette*, Dec. 6, 1856.

YORK, November 5, 1856.

7. A PATULOUS STATE OF THE OS UTERI, AND ITS RELATION TO ABORTION.—At a late meeting of the Western Medical and Surgical Society of London, Mr. Ellis, after remarking upon the frequency of abortion, and its effects upon the health of the patients, alluded to the habit into which the uterus falls of casting off its contents prematurely, and mentioned one case in which the patient aborted 27 times. The causes of abortion may be ovaline or maternal, the latter being impoverished health, leucorrhœa, uterine irritation, or irritation caused from the bladder or rectum. But he alluded more especially to a patulous state of the os uteri as a cause, in which the proper natural plug from the decidua was absent.

In these cases the os uteri was open, and readily allowed the bag of membranes to protrude, and as surely as they did so, so certainly would the uterus be excited and contract. An analogous case was seen where premature labor was induced by the introduction of a piece of sponge into the upper portion of the vagina, and which by its dilatation had the effect of opening the os uteri. The causes of this state, he considered to result from inflammation, and structural changes, and in its treatment, recommended rest, and the occasional use of nitrate of silver to the patulous os uteri.—*Ibid.*

8. RULES FOR THE RESUSCITATION OF STILL-BORN INFANTS.—Dr. Marshall Hall also proposes that the plan which he has recommended for the recovery of the apparently drowned should be employed for the resuscitation of infants born at maturity, but apparently dead. The following are his "rules;" we re-publish them from the Lancet, of November 29th (p. 601):

"1. The infant must be placed in the prone position, in order that all fluids, which might obstruct the entrance into the windpipe, may flow away.

"2. Nature's mode of operation being to impress the trifacial and cutaneous nerves, the external *excitors* of respiration, by the external cold, we must dash a few drops of cold water on the face and the general surface.

"3. We must proceed, having failed to *excite* respiration, to *imitate* the respiratory movements:

"This must not be done by *any forcing* means; even the human breath, forced into the infant's lips, may *tear* the delicate tissue of the fœtal lungs. We must, on the contrary, adopt some measure of *drawing* the air into the lungs. This is effectually accomplished by first placing the little patient briskly in the prone position, to clear the fauces; then pressing gently on the back; and then removing that pressure, and turning it gently on the side and a little beyond.

"4. Meantime, the limbs are to be rubbed, with gentle pressure, upwards, to promote the circulation, by propelling the venous blood towards the heart.

"5. At proper intervals, we must again endeavor to *excite* the respiration physiologically.

"The infant is to be placed with the face prone, and douched alternately and rapidly with water of the temperatures of 60° and 100° Fahr.

"High and low temperatures are equally excitants of the reflex function of respiration, and their power, within physiological limits, is in proportion to the difference of those temperatures."

The successful application of these rules may be already announced, as will be seen by the following letter addressed by Mr. J. T. WALLER to Dr. MARSHALL HALL:—

FLEGG BURGH, NORFOLK, Dec. 4, 1856.

DEAR SIR:—I was so delighted with a report in the Lancet of Nov. 29th, of a paper read by you before the Harveian Society, on the Asphyxia of Infants and the Treatment, that I hope you will excuse the liberty I have taken in sending the following brief details. I trust you remember me as a pupil of yours at Webb Street, some twenty years ago.

On Tuesday, the 23d ult., at seven P. M., I was called to Mrs. P——, aged forty-five, in labor with her first child. On the 24th, at six A. M., I delivered her, with a forceps, of a very fine female child, but in a state of complete asphyxia. Your "Ready Method" was adopted. As soon as I had tied the cord, I exposed the child freely to the cool air, cleared the mouth and nostrils, turned the face and chest downwards, and began the alternate movements of the body on to the side and face, repeating them every ten seconds; blowing the cold air on the child with the bellows, alternately with the application of flannels dipped in hot water to the chest. *For a quarter of an hour the case appeared quite hopeless;* soon afterwards, the infant began to respire, about once in a minute; and from that time it improved gradually, so that in about *two hours* it was perfectly recovered.

After reading your paper on the subject, and this confirmation of your views, I shall feel quite satisfied in pursuing this course to the exclusion of all others—viz., the hot-bath, and inflation of the lungs with a tube or the mouth, with which I was never very successful; and you may be sure that I have had no want of experience when I inform you that I attend on an average more than a hundred labors in one year.

I am, dear Sir, truly yours,

JOHN T. WALLER.

MARSHALL HALL, Esq., M.D., F.R.S.,
London.

*Lancet*, Dec. 13, 1856.

